# Multi-Agent Reinforcement Learning-Based Computation Offloading for Unmanned Aerial Vehicle Post-Disaster Rescue

**DOI:** 10.3390/s24248014

**Published:** 2024-12-15

**Authors:** Lixing Wang, Huirong Jiao

**Affiliations:** School of Computer Science and Engineering, Northeastern University, Shenyang 110000, China; 2201807@stu.neu.edu.cn

**Keywords:** mobile edge computing, computation offloading, unmanned aerial vehicle, post-disaster rescue, multi-agent reinforcement learning

## Abstract

Natural disasters cause significant losses. Unmanned aerial vehicles (UAVs) are valuable in rescue missions but need to offload tasks to edge servers due to their limited computing power and battery life. This study proposes a task offloading decision algorithm called the multi-agent deep deterministic policy gradient with cooperation and experience replay (CER-MADDPG), which is based on multi-agent reinforcement learning for UAV computation offloading. CER-MADDPG emphasizes collaboration between UAVs and uses historical UAV experiences to classify and obtain optimal strategies. It enables collaboration among edge devices through the design of the ’critic’ network. Additionally, by defining good and bad experiences for UAVs, experiences are classified into two separate buffers, allowing UAVs to learn from them, seek benefits, avoid harm, and reduce system overhead. The performance of CER-MADDPG was verified through simulations in two aspects. First, the influence of key hyperparameters on performance was examined, and the optimal values were determined. Second, CER-MADDPG was compared with other baseline algorithms. The results show that compared with MADDPG and stochastic game-based resource allocation with prioritized experience replay, CER-MADDPG achieves the lowest system overhead and superior stability and scalability.

## 1. Introduction

Unmanned aerial vehicles (UAVs) are widely used in various fields, such as crime scene surveillance [1], habitat destruction assessment [2], crop monitoring [3], and vegetation mapping [4]. Recently, UAVs have been used in humanitarian rescue operations [5]. UAVs can be combined with artificial intelligence technology to improve the efficiency of natural disaster rescue efforts. UAVs are characterized by their low cost, high maneuverability, and flexible deployment, allowing them to easily enter disaster-stricken areas that are difficult to reach otherwise [6]. UAVs use onboard sensors and wireless communications to create digital maps of post-disaster areas and can be used as communication relays to effectively forward scheduling and commands of post-disaster rescue to ground rescue equipment [7]. Recent work has investigated the age of information (AoI)-minimal clustering, transmission, and trajectory co-design in UAV-assisted wireless-powered communication networks (WPCNs). In these networks, UAVs with limited battery capacity must manage energy-efficient clustering of islands and optimize their flight trajectories to ensure effective data transmission and long-term performance [8].

The use of UAVs in post-disaster rescue operations is subject to fundamental engineering challenges. On the one hand, UAVs have strict weight requirements, limiting the capacity of their onboard batteries [9]. On the other hand, some tasks have deadlines, and because of the limited computational capacity of UAVs, real-time applications, such as image processing and video streaming, often exceed the local data-processing capacity of UAVs. Therefore, UAVs must offload a large number of intensive computing tasks to nearby edge servers during post-disaster rescue to respond to rescue efforts in a timely manner and extend their lifecycle. These studies highlight the importance of designing intelligent offloading strategies to balance energy consumption and task performance in UAV-assisted systems. UAVs must consider the weighted sum of the task completion time and system energy consumption as the system overhead, and the goal of the joint offloading strategy is to minimize system utilization. Our motivation is to optimize the UAV’s computation offloading decisions to reduce system overhead. Minimizing system overhead can improve the overall task completion efficiency. System overhead includes both time and communication overheads. Therefore, the means to improve the overall task completion efficiency is to minimize time consumption and communication consumption, achieving the highest efficiency for completing computation offloading tasks. The use of multi-agent reinforcement learning methods to determine optimal offloading strategies for UAV computation offloading scenarios has emerged as a topic of significant research interest [10].

In this study, a mobile edge computing offloading system called the multi-agent deep deterministic policy gradient with cooperation and experience replay (CER-MADDPG) with multiple UAVs and servers is proposed, in which UAVs generate tasks with delay constraints and continuously send offloading requests to edge servers during their movement. We define the trade-off between the delay and energy consumption as the system overhead. To minimize the system overhead caused by the decisions made by the UAVs, a swarm of UAVs makes joint offloading decisions using CER-MADDPG. The main contributions of this study are summarized as follows:

- The UAV swarm makes joint offloading decisions by modeling the offloading environment, delegating offloading decision-making to UAVs, and considering the random mobility of edge server clusters, time-varying nature of channels, and signal blockage of UAVs. To avoid wasting server computing resources while ensuring the successful execution of user tasks, the system is modeled as an optimization problem that minimizes the system overhead using the weighted average of the task execution time and server energy consumption as the system overhead.
- The CER-MADDPG algorithm based on multi-agent reinforcement learning is proposed. The UAVs use this algorithm to obtain their next coordinates, select edge servers, and determine the task offloading ratios. The algorithm enhances the collaborative decisions between agents by considering both global and individual information. Additionally, the algorithm classifies historical experiences and learns according to different categories of experiences, allowing UAVs to fully utilize historical experiences in continuous decision-making, cooperation, and optimal joint decisions.
- Experiments were conducted using the PyTorch platform to simulate the proposed algorithm and compare it with MADDPG and stochastic game-based resource allocation with prioritized experience replays (SGRA-PERs), thereby verifying that the proposed algorithm is superior to other algorithms in terms of optimality, stability, and scalability.

The remainder of the article is organized as follows: Section 2 reviews relevant related work. Section 3 describes the UAV edge server problem. Section 4 introduces an offloading decision model based on the CER-MADDPG algorithm. Section 5 presents an analysis of the performance of the CER-MADDPG algorithm using simulations, and Section 6 presents the conclusions.

## 2. Related Work

Computation offloading is a core issue in edge computing. Zhang et al. [11] argued that the main solutions to computation offloading are tasks to be offloaded and offloading locations. Current offloading decisions often involve comprehensive delay, energy consumption, or user benefits as offloading objectives to meet the needs of real-time and low energy consumption.

Because finding an optimal solution to the computation offloading problem is difficult, several studies have proposed algorithms based on searching for suboptimal solutions, such as metaheuristic-based algorithms. Among these, the genetic algorithm is most widely used, which has low computational cost and can obtain solutions very close to the optimal solution in some cases. Li et al. [12] used the genetic algorithm to solve a scenario with multiple mobile devices and one edge server, dividing the tasks of edge devices proportionally to minimize the overall task completion time. Al-habob et al. [13] modeled the computation offloading scenario as an optimization problem with binary scheduling decision variables and designed a genetic algorithm to solve the optimization problem.

Another approach for solving optimization problems is based on deep reinforcement learning algorithms. Hu et al. [14] modeled the offloading problem as a Markov decision process and designed an offloading strategy based on deep deterministic policy gradient to dynamically adjust the offloading ratio, maximizing the system overhead considering server energy consumption and task duration. Huang et al. [15] proposed an online offloading framework based on deep reinforcement learning, optimizing the task offloading decision of a single wireless device according to the time-varying wireless channel. Yang et al. [16] divided the offloading decision into discrete actions, jointly deciding on task offloading, wireless channel allocation, and image compression rate selection to achieve higher average image recognition accuracy and lower average processing latency.

In some scenarios, single-agent reinforcement learning methods are not applicable. For example, in large-scale disaster relief, UAVs have different sensors and capabilities. Multi-agent reinforcement learning allows multiple agents to make independent decisions, with each agent responsible for solving specific sub-problems, thus better adapting to the decomposition and decision-making of complex tasks. Nguyen et al. [17] used MADDPG for joint data offloading in multiple independent edge clouds. Peng et al. [18] used the MADDPG method to solve the decision-making problem of UAV computation offloading with the goal of maximum reduction in the number of tasks. Lu et al. [19] considered minimizing the task failure rate and improving system utility as objectives to solve the task offloading problem of edge devices, based on the MADDPG algorithm. Huang et al. [20] proposed the cost-aware collaborative task-execution model, which specifically considers long-term reward indicators such as the number of lost tasks and uses the MADDPG method to coordinate multiple energy harvesting mobile devices in an EH-D2D network to execute computing tasks. Kumar et al. [21] treated each vehicle as an intelligent agent with decision-making capabilities and classified tasks generated by vehicles into local execution queues or task offloading queues using a method known as the Lyapunov-based MADDPG, which can minimize energy consumption while maintaining queue stability. However, these studies have shortcomings considering the cooperation between edge devices, which can lead to suboptimal joint decisions made by a group of edge devices.

Insufficient experience can lead to a system that does not achieve an optimal solution. Generally, reinforcement learning algorithms require a series of iterations to achieve the optimal solution. Argerich et al. [22] proposed using external knowledge to guide agent decisions, which improves performance during training using expert knowledge from a database in the form of a programmable function. Chen et al. [23] stored the historical experiences of edge devices in a priority experience buffer, repeatedly learning from key historical experiences to improve the learning efficiency and obtain higher-quality solutions. Wu et al. [24] prioritized the historical experiences of each UAV in a tree-like manner, repeatedly learning from less frequent but important experiences to improve the system performance. However, these methods have shortcomings in the utilization of historical experiences of edge devices, which is also one of the reasons why the joint decisions made by a group of edge devices fall into local optima.

In recent years, with the widespread application of unmanned aerial vehicles (UAVs) in fields such as post-disaster rescue and environmental monitoring, multi-UAV and multi-MEC (mobile edge computing) collaborative systems have become a hot research topic. Multi-UAV systems are capable of providing flexible computational and communication resources, particularly in scenarios where traditional cloud and edge computing capabilities are limited, effectively compensating for the lack of computational resources. Asif et al. [25] proposed a Joint Data Aggregation and Computation Offloading (JDACO) scheme, utilizing multiple UAVs as MEC servers to optimize data aggregation and task offloading processes, minimizing energy consumption and latency, showcasing UAVs’ potential in providing computational support for IoT devices in post-disaster scenarios. Zhang et al. [26] introduced a device-edge-cloud collaborative computing model that optimizes system delay and energy consumption, addressing the limited computational power of UAVs. The study employs a SAC-based reinforcement learning algorithm (STS-UDCO) to improve system convergence speed and stability, offering an effective solution for multi-UAV computation offloading. Wang et al. [27] presented a multi-UAV-assisted MEC system, incorporating Reconfigurable Intelligent Surfaces (RIS) to enhance communication performance and reduce system delay. By optimizing the computation offloading strategy and UAV trajectory, and employing a multi-agent deep reinforcement learning algorithm (MATD3), the study significantly improves system fairness and delay performance. Although these studies provide valuable insights into multi-UAV and multi-MEC systems for post-disaster rescue and resource allocation, they share a common limitation. While collaboration mechanisms and task offloading optimization are addressed in each study, they do not fully consider the integration of collaboration with the effective utilization of historical experiences. Specifically, existing approaches typically focus on optimizing a single objective, neglecting the potential of using intelligent experience replay mechanisms and collaborative decision-making to enhance overall system performance.

Although several studies have attempted to use multi-agent reinforcement learning methods to solve the UAV computation offloading problem, we found the following:

1. Most current works consider scenarios with a single server and multiple UAVs, whereas real scenarios often involve multiple servers and multiple UAVs.
2. Existing research does not fully consider cooperative decision-making between UAVs. For example, when multiple UAVs observe the same environmental state in the same time slot, they make the same decisions. This homogenized decision-making is not necessarily a joint optimal decision, which may lead to joint offloading decisions made by a group of UAVs to fall into local optima.
3. Existing studies have not sufficiently considered the utilization of historical UAV decision-making experiences. Current research focuses on prioritizing historical UAV experiences or introducing external knowledge bases. The UAV group cannot learn quickly from high-quality historical decision-making experiences and avoid poor experiences. This can also lead to joint offloading decisions made by a group of UAVs to fall into local optima.

As discussed in this section, several architectures and technologies exist to solve the computation offloading problem, each with its own advantages and disadvantages. Among these, technology based on multi-agent reinforcement learning is the most promising. Therefore, we propose a UAV edge-computing offloading method based on multi-agent reinforcement learning, aiming to jointly optimize the task completion time and server energy consumption. Existing studies do not fully consider cooperative decision-making between UAVs or the full utilization of historical UAV decision-making experiences, resulting in suboptimal system overhead. To address these shortcomings, we propose the CER-MADDPG algorithm, which uses a neural network design to achieve cooperative decision-making between UAVs and guides the joint decision-making of a UAV group toward an optimal solution through the classification of historical UAV decision-making experiences.

## 3. Problem Description

In this section, we introduce the UAV computation offloading system. The system is divided into the UAV and edge layers, and the movement of devices in both layers is modeled with the devices able to move in their respective layers. Information exchange can occur between UAVs and edge devices. The system comprises three sub-models of mobile edge computing: network communication, task computation, and task transfer.

### 3.1. MEC Environment Model

**Figure 1:**
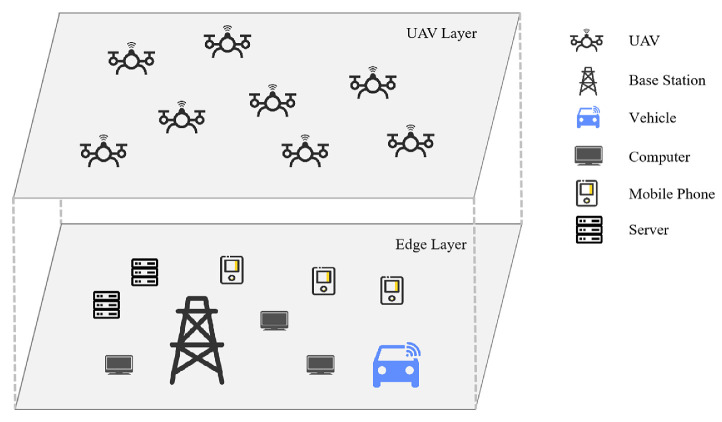
Edge computing architecture.

The system comprises two layers of devices, as shown in Figure 1, namely the UAV layer and edge layer. In this structure, the devices in the edge layer provide computing resources for the UAV group, and the UAVs can fly within the system architecture. Our work focuses on effective offloading decisions of UAVs. Let the set of UAVs be N={1,2,⋯,n} and the set of edge devices be M={1,2,⋯,m}. After each UAV randomly generates a task, it determines the distance and angle of flight and offloading ratio of the task and selects an edge device to send the offloading request in each time interval. The corresponding device performs the offloading computations. The main components of the MEC environment model include the UAV mobility, edge server mobility, and UAV task generation models, as described below.

#### 3.1.1. UAV Mobility Model

Let the flight angle and flight distance of the UAV be θ and dist, respectively, and the current coordinates of the UAV be $\left(uav_{x},uav_{y}\right)$. The position of the UAV in the next time slot is given by (1):

(1) $$\begin{array}{cc} uav_{x} & =uav_{x}+dist\cdot \cos\left(\theta \right)\\ uav_{y} & =uav_{y}+dist\cdot \sin\left(\theta \right) \end{array}$$

The energy consumption battery of the UAV flight to the next destination is given by (2).

(2) $$battery=battery-r\cdot f_{uav}^{2}\cdot x\cdot taskSize\cdot s$$

where $f_{uav}$ represents the computation frequency of the UAV, *x* represents the computation offloading ratio, taskSize represents the size of the UAV task, *s* represents the number of CPU cycles required to process one bit, and *r* represents the impact factor of the chip structure on the CPU processing.

#### 3.1.2. Edge Server Mobility Model

The current coordinates of the edge server are $\left(ue_{x},ue_{y}\right)$. At each time step, it randomly moves one step. The possible coordinates of the edge server in the next time step are as follows:

$$\left\{\begin{array}{c} \left(ue_{x}-1,ue_{y}\right)\\ \left(ue_{x}+1,ue_{y}\right)\\ \left(ue_{x},ue_{y}-1\right)\\ \left(ue_{x},ue_{y}+1\right)\\ \left(ue_{x},ue_{y}\right) \end{array}\right.$$

#### 3.1.3. UAV Task Generation Model

Each UAV generates task $T_{n}$ in the initial state, including the task generation time, expiration time, and task size. In each time slot, each UAV selects an angle and distance to move and an edge server for the partial offloading of tasks.

### 3.2. Communication Model

We assume that the wireless channel bandwidth for transmission from the UAV to the edge server is *B* Hz. Additionally, the transmission loss power is defined as $p_{noisy}$, and the reference channel gain when the distance between UAV and edge server is 1 m is defined as $\alpha_{0}$. We denote the uplink transmission power as $p_{uplink}$. The distance between the UAV and selected edge server was calculated based on the positions of the UAV and edge server as dist(uav,ue).

The channel gain g(uav,ue) and uplink transmission rate transRate are given by Formulas (3) and (4), respectively.

(3) $$g\left(uav,ue\right)=\left|\frac{\alpha_{0}}{\mathrm{dist}{\left(uav,ue\right)}^{2}}\right|$$

(4) $$\begin{array}{c} transRate=B\cdot \log_{2}\left(1+\frac{p_{uplink}\cdot g\left(uav,ue\right)}{p_{noisy}}\right) \end{array}$$

The communication delay $t_{tr}$ and communication energy consumption $e_{tr}$ are given by Formulas (5) and (6), respectively, where taskSize represents the task size of UAV *n*, *x* represents the task offloading ratio, transRate is the uplink transmission rate obtained from (4), and $P_{1}$ represents the power consumed for communication computation.

(5) $$t_{tr}=x\cdot \frac{taskSize}{transRate}$$

(6) $$e_{tr}=x\cdot \frac{taskSize}{transRate}\cdot P_{1}$$

### 3.3. Computation Offloading Model

#### 3.3.1. Local Model

The tasks selected for local computation are provided with computing resources by the UAV. The local computation delay $t_{local}$ and local energy consumption $e_{local}$ are given by Formulas (7) and (8), respectively.

(7) $$t_{local}=\left(1-x\right)\cdot \frac{taskSize}{f_{uav}\cdot s}$$

(8) $$e_{local}=\left(1-x\right)\cdot \frac{taskSize}{f_{uav}\cdot s}\cdot P_{2}$$

where *x* is the offloading ratio, taskSize represents the task size of UAV *n*, $f_{uav}$ is the computing frequency of the UAV, *s* is the number of CPU cycles required to process one bit of the task, and $P_{2}$ represents the power consumed for local computation.

#### 3.3.2. Server Model

The tasks selected for server computation are provided with computing resources using an edge server. The server computation delay $t_{edge}$ and server energy consumption $e_{edge}$ are given by Formulas (9) and (10), respectively.

(9) $$t_{edge}=x\cdot \frac{taskSize}{f_{uav}\cdot s}$$

(10) $$e_{edge}=x\cdot \frac{taskSize}{f_{uav}\cdot s}\cdot P_{3}$$

Therefore, the total time consumption $t_{com}$ is given by Formula (11), and the total energy consumption $e_{com}$ is given by Formula (12), where *x* is the offloading ratio, taskSize represents the task size of UAV *n*, $f_{ue}$ is the computing frequency of the edge server, *s* is the number of CPU cycles required to process one bit of the task, and $P_{3}$ represents the power consumed for the server computation.

(11) $$t_{com}=\max\left(t_{tr}+t_{edge},t_{local}\right)$$

(12) $$e_{com}=e_{tr}+e_{edge}+e_{local}$$

The objective of the optimization problem for the optimal offloading decision is to minimize the system cost through rational decision-making of the UAV fleet. The system cost comprises the total time consumption $t_{com}$ and total energy consumption $e_{com}$. The specific optimization problem is shown in Formula (13).

$$\begin{array}{cc} \mathrm{Minimize}\; & D=\sum_{i=1}^{N}[\lambda_{1}\cdot \max\left(t_{tr_{i}}+t_{edge_{i}},t_{local_{i}}\right) \end{array}$$

(13) $$\begin{array}{cc} \; & +\lambda_{2}\cdot \max\left(e_{tr_{i}}+e_{edge_{i}},e_{local_{i}}\right)] \end{array}$$

(13a) $$\begin{array}{cc} \mathrm{subject}\;\mathrm{to}\; & \max\left(t_{tr_{i}}+t_{edge_{i}},t_{local_{i}}\right)<T_{\max_{i}} \end{array}$$

(13b) $$\begin{array}{cc} \; & x(i)\in [0,1] \end{array}$$

Here, $\lambda_{1}$ represents the hyperparameter related to the total time consumption, and $\lambda_{2}$ represents the hyperparameter related to the total energy consumption. Formula (13a) indicates that the tasks processed by the UAV should not exceed the deadline, and Formula (13b) indicates that the offloading ratio should be between 0 and 1.

## 4. Multi-Agent Deep Deterministic Policy Gradient Considering Collaboration and Experience Utilization

In this section, we will define the UAV computation offloading system designed in Section 3 using the partially observable Markov decision process (POMDP). In addition, we provide a detailed introduction to the improved MADDPG (CER-MADDPG) model and explain its training process.

### 4.1. Partially Observable Markov Decision Process

Each UAV can be considered an intelligent agent with decision-making capabilities, and we train an offloading strategy for each UAV. The POMDP is defined as (S,A,R,P,O), where *S* represents the set of states, *A* the set of actions, *R* the reward function, *P* the state transition probability, and *O* the set of all joint observation values of the agents.

State: At time slot *t*, the state $s_{t}^{i}$ of UAV *i* includes the characteristics of the UAV $N_{n}$ and the characteristics of the base station $D_{m}$. $N_{n}$ includes the remaining battery level battery, the coordinates of the UAV $\left(uav_{x},uav_{y}\right)$, and the flight speed of the UAV speed. $D_{m}$ includes the current CPU usage cpu, number of tasks currently executed tr, maximum computing capability of the processor mips, energy consumption per bit for the transmission ee, and bandwidth of the edge device eb. The specific content is shown in Formula (14).

(14) $$s_{t}^{i}={\left\{N_{i},D_{j}\right\}}_{i=1,j=1}^{N,M}$$

The state of the entire system contains the states of all UAVs, denoted by $S_{t}$, as shown in (15).

(15) $$S_{t}={\left\{s_{t}^{i}\right\}}_{i=1}^{N}$$

Action: The decision $a_{t}^{i}$ made by UAV *i* at time *t* includes the selection of the edge server *m*, next flight angle angle, distance dist, and offloading ratio for computation *x*, as shown in Formula (16).

(16) $$a_{t}^{i}=\left\{m,angle_{i},dist_{i},x\right\}$$

The action set of each agent forms the action space $A_{t}$ of the entire system, as expressed in Formula (17).

(17) $$A_{t}={\left\{a_{t}^{i}\right\}}_{i=1}^{N}$$

Reward: Referring to Formula (13), the reward reward considers both the completion time and energy consumption of the same batch of tasks. The reward is defined as shown in Formula (18).

(18) reward=−D

Transition Probability: In a continuous action space, state transition is directly caused by the actions of the UAVs. As shown in Formula (19), the deterministic policy μ is used in this paper because of its efficiency, which is tens of times higher than that of a random policy, significantly reducing the training time.

(19) $$a_{t}^{i}=-\left(s_{\;t}^{\prime i}\right)$$

Observation: The information that UAV *i* can perceive is represented by observation $o_{t}^{i}$ of UAV *i*, as shown in Formula (20). UAV *i* can only observe information from its local environment and partial base stations.

(20) $$o_{t}^{i}={\left\{N_{i},D_{j}\right\}}_{j=1}^{k}$$

The observation set $O_{t}$ is shown in Formula (21).

(21) $$O_{t}={\left\{o_{t}^{i}\right\}}_{i=1}^{N}$$

Cumulative Return: The return $r_{\mathrm{sum}}$, as shown in Formula (22), is the accumulation of rewards over time, which is the sum of all rewards on the time axis. The goal of this study was to maximize the returns within each time period.

(22) $$r_{sum}=\sum_{i=1}^{N}\gamma^{N-i}r_{i}$$

### 4.2. Multi-Agent Deep Deterministic Policy Gradient Considering Collaboration and Experience Utilization

#### 4.2.1. General Description

Based on the model described in the previous section, this study proposes an improved MADDPG algorithm (CER-MADDPG) to solve the optimization problem described in (13). First, the CER-MADDPG algorithm considers cooperative decision making among multiple UAVs. Second, the CER-MADDPG algorithm focuses on guiding the UAVs’ decisions toward good experiences and away from bad ones. When the UAVs are in the same state, they may perform homogeneous actions, which reduces the overall efficiency of the system. Therefore, collaborative decision-making among UAVs is necessary. In addition, the traditional priority-experience buffer does not fully utilize these experiences. Therefore, this study considers categorizing experiences to make the decisions of the UAV group closer to good experiences and further from bad ones.

In this study, the proposed CER-MADDPG algorithm is based on the partial offloading model. Compared to the full offloading model, partial offloading offers greater flexibility and efficiency in task processing. While full offloading alleviates the UAV’s computational burden by offloading all tasks to the edge server, it fails to fully utilize the local computation resources of the UAV, potentially leading to unnecessary network transmission delays and bandwidth wastage. In contrast, partial offloading allows UAVs to flexibly allocate tasks between local computation and edge servers based on the specific requirements of the tasks, thereby optimizing the balance between computational capacity and energy consumption. By dynamically adjusting the ratio of local computation and offloading, partial offloading not only reduces network transmission load but also minimizes system overhead, improving task processing efficiency and real-time performance. Additionally, partial offloading can extend the UAV’s flight time to some extent, as UAVs can decide whether to keep part of the computation locally based on task complexity, thus reducing reliance on battery power.

**Figure 2:**
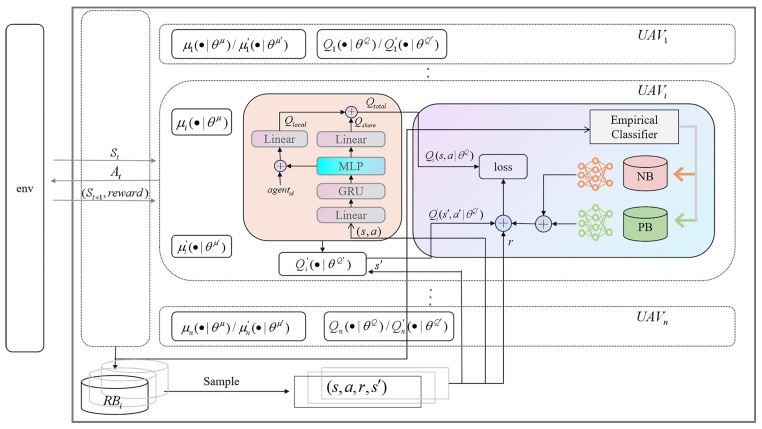
CER-MADDPG algorithm structure.

CER-MADDPG comprises multiple collaborating UAVs, each comprising an evaluation network, target network, and replay buffer $RB_{i}$. The evaluation network includes actor evaluation networks $\mu_{i}\left(•|\theta^{\mu }\right)$ and critic evaluation networks $Q_{i}\left(•|\theta^{Q}\right)$. The target network includes actor target networks $\mu_{i}^{\prime }\left(•|\theta^{\mu^{\prime }}\right)$ and critic target networks $Q_{i}^{\prime }\left(•|\theta^{Q^{\prime }}\right)$. Here, $\theta^{\mu }$, $\theta^{Q}$, $\theta^{\mu^{\prime }}$ and $\theta^{Q^{\prime }}$ represent the parameters of the networks. As shown in Figure 2, at time *t*, all UAVs observe the state $S_{t}$ and input it into the action network. By calculating the policy function, the action vector $A_{t}$ is obtained. Subsequently, the state is updated to $S_{t+1}$, and a reward is generated. The observed state of the UAV, action vector of the UAV, reward feedback from the environment, and next state of the environment are stored in the UAV’s respective experience replay buffer $RB_{i}$ in the form of $\left(s,a,r,s^{\prime }\right)$ tuples. A batch of experiences $\left(s,a,r,s^{\prime }\right)$ is extracted from the experience replay buffer, and state *s* and action *a* are input into the critic network. The Q value is calculated through $Q_{i}\left(•|\theta^{Q}\right)$ to describe whether the action is appropriate in the current state.

Joint actions *a* determine the next state and reward. The goal of the system is to find the optimal policy $\pi^{*}\left(s\right)=\arg\max_{\pi }Q_{n}^{\pi }\left(s,a\right)$ that can select the optimal action in the current state to maximize the expected total discounted reward in the future. We use $Q_{i}\left(s,a|\theta^{Q}\right)$ and $Q_{i}\left(s^{\prime },a^{\prime }|\theta^{Q}\right)$ to represent the Q-values at the current action state and Q-values at the next action and state, defined as (23):

(23) $$Q_{\mu }\left(s,a\right)=\left\{\sum_{t=0}^{\infty }\gamma_{k}R^{t+1}|s,a\right\}$$

#### 4.2.2. Critic Networks That Consider Both Global and Local Information

**Figure 3:**
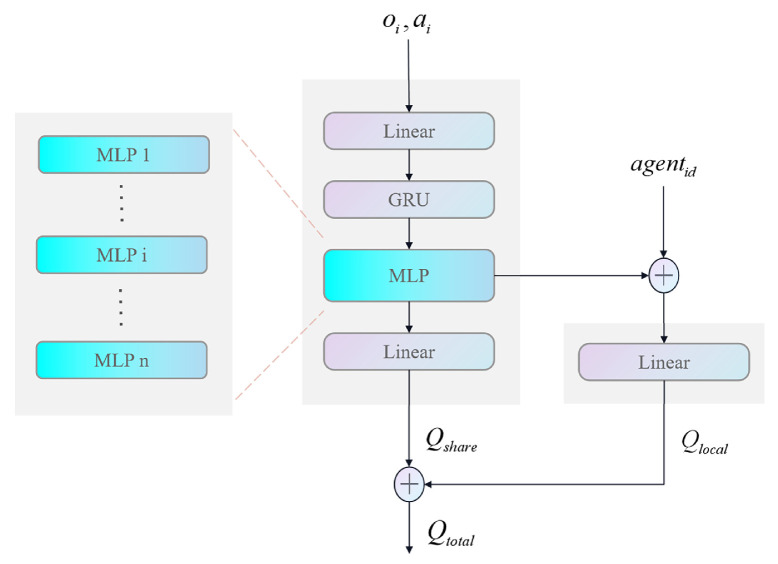
Improved critic network structure.

Here, we design the $Q_{i}\left(•|\theta^{Q}\right)$ network structure in a parallel form, as shown in Figure 3. To avoid the UAV group taking the same actions when facing the same environmental state, which may lead to suboptimal system utility, we integrated the individual information of the UAVs into the neural network. The network was designed using shared and individual layers. The shared part of the neural network is used to process the global information and obtain $Q_{share}$. In addition, a separate neural network was designed for each agent to process the individual information, and the individual information of the agent was concatenated to obtain $Q_{local}$. This approach helps balance the processing of global and individual information to some extent. Finally, $Q_{share}$ and $Q_{local}$ are concatenated to obtain the final value $Q_{total}$, which guides the update of the actor network.

Here, γ is the discount factor, which represents the importance of future states for the current state. The parameter update of the evaluation network depends on the TD error, and $\theta^{Q}$ is updated by minimizing the loss function. The loss function can be expressed as (24):

(24) $$\begin{array}{cc} L(\theta^{Q})= & \frac{1}{{\mathrm{sum}}_{i}}\sum [\left(R^{i}+\gamma Q^{\prime }\left(S^{i+1},\mu^{\prime }\left(S^{i+1}|\theta^{\mu^{\prime }}\right)|\theta^{Q}\right)\right)\\ \; & -Q\left(S^{i},a^{i}|\theta^{Q}\right){]}^{2} \end{array}$$

Here, $sum_{i}$ represents the total number of UAVs, and the critic network must be continuously optimized during each iteration of the training process to minimize the loss function. To ensure computational efficiency, a batch gradient descent was used to optimize the loss function and update the weight parameters. The parameter update of the actor network depends on the output of the critic network, which trains the policy network by maximizing the Q-value estimate of the critic network for the action output by the actor network. The update of the actor network parameters according to the deterministic policy gradient ascent strategy can be expressed as (25):

(25) $$\nabla_{\theta^{\mu }}J\left(\mu \right)\approx \frac{1}{{\mathrm{sum}}_{i}}\sum \left(\nabla_{a}Q\left(S^{i},a^{i}|\theta^{Q}\right)\nabla_{\theta^{\mu }}\mu \left(S^{i}|\theta^{\mu }\right)\right)$$

During the training process, exponential smoothing is used to update the two target networks. The updating method for the target network parameters can be expressed as (26):

(26) $$\begin{array}{c} \theta^{Q^{\prime }}\leftarrow tau\theta^{Q}+\left(1-tau\right)\theta^{Q^{\prime }}\\ \theta^{\mu^{\prime }}\leftarrow tau\theta^{\mu }+\left(1-tau\right)\theta^{\mu^{\prime }} \end{array}$$

Here, the parameter 0<θ≪1 is used to ensure that the target networks update slowly and steadily, improving the stability of learning.

#### 4.2.3. Utilization of Categorized Experience

**Figure 4:**
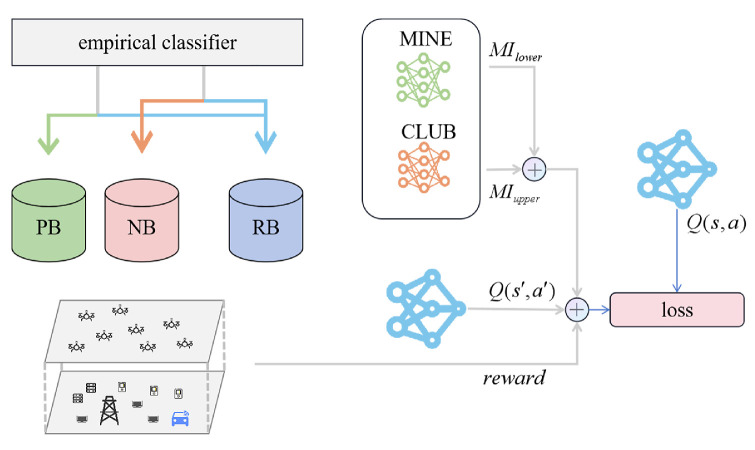
Good and bad behavior guidance model.

The UAVs’ experiences generated in this process are stored in their respective experience replay buffers (RB). To better utilize the UAVs’ experiences, Figure 4 describes the categorization of the UAV group’s experiences, guiding them toward joint actions that are beneficial and avoiding harmful ones.

$I(S_{t},A_{t})$ represents the mutual information between the global state and the joint decision, which can describe the relevance between the global state and decisions. The larger $I(S_{t},A_{t})$ is, the higher the correlation between the global state and joint decision, indicating that both high- and low-quality joint actions may have a high correlation with the environment. In this study, a bidirectional guidance module was designed to increase the correlation between high-quality joint actions and their corresponding global states while reducing the correlation between low-quality joint actions and their corresponding global states. The mutual information neural estimation (MINE) and contrastive log-ratio upper bound (CLUB) networks are used to estimate the correlation between two random variables. The MINE network [28] is used to estimate the lower bound of $I(S_{t},A_{t})$, and the CLUB network [29] is used to estimate the upper bound of $I(S_{t},A_{t})$. Let $MI_{lower}$ be the value estimated by the MINE network, and $MI_{upper}$ be the value estimated value provided by the CLUB network. The values from both neural networks were integrated into the reward to train the policy selection of the UAV network. Increasing the lower bound of the high-quality experience buffer and decreasing that of the low-quality experience buffer can promote collaboration among UAVs, thereby achieving the goal of reducing system utility. The reward was supplemented as shown in the following Formula (27).

(27) $$reward=-D-\alpha_{1}\cdot MI_{upper}+\alpha_{2}\cdot MI_{lower}$$

The method proposed in this paper for classifying good and bad experiences is as follows. An experience is considered a high-quality experience and enters the high-quality experience buffer PB if its reward is greater than the worst experience in the buffer $PB_{low}$ and its reward exceeds the average reward $\bar{r}$ over a period of time. Otherwise, the experience enters the buffer NB. Regardless of whether the experience is good or bad, it enters the priority buffer RB for extraction and learning during UAV model training. The determination of a high-quality experience is shown in the following Formula (28).

(28) $$r_{sum}>\max\left(\bar{r},PB_{low}\right)$$

The experiences of the UAV swarm are classified by the classifier, with high-quality experiences entering PB and poor-quality experiences entering NB. Both high- and poor-quality experiences enter the general experience buffer, RB. Experiences in the high-quality experience buffer PB were used to train the MINE network, whereas experiences in the poor-quality experience buffer NB were used to train the CLUB network. The overall reward for training the critic network comprises both the environmental reward and estimated mutual information value.

### 4.3. Training Process

The training process using CER-MADDPG to solve the UAV computation offloading problem proposed in this study is as follows:

The Algorithm 1 initialization is described in lines 1–3. First, the action networks, value networks, MINE, and CLUB were initialized for each UAV. Additionally, the experience buffers PB, RB, and NB, and finally, the positions of the UAVs and edge devices are initialized. Training starts in line 4, where each UAV observes its state $s_{n}$ from the environment. Each UAV obtains an action from the action network based on its current state. The UAVs then execute joint actions and receive the current reward $r_{n}$ and the next state $s_{n}^{\prime }$. The $\left(s,a,r,s^{\prime }\right)$ tuples of the UAVs are stored in their respective experience replay buffers, RB. The experiences of the UAV swarm are classified by the classifier to determine whether to store them in the high-quality experience buffer PB or the low-quality experience buffer NB. When there are sufficient experiences with RB, a small batch is sampled from the replay buffer. Based on this batch, the critic and actor networks are updated. When there are sufficient experiences in both PB and NB, experiences are extracted from both to update the MINE and CLUB networks.
**Algorithm 1** The CER-MADDPG Training Procedure in the UAV Computation Offloading System**Require:** Replay buffer RB, positive buffer PB, negative buffer NB, time budget *T*, exploration probability ε, discount factor γ, update step ξ

**Ensure:** The optimal policy $\pi_{\theta_{n}}^{*}$
1:**Initialize** actor and critic networks for each UAV, MINE and CLUB.2:**Initialize** experience replay buffer RB, PB, and NB.3:**Initialize** the location of UAVs and edge servers.4:**for** episode=1 to *M* **do**5:       Initialize the state $s0\leftarrow {\left\{N_{i},D_{j}\right\}}_{i=1,j=1}^{N,M},t_{0}$6:   **for** t=1 to *T* **do**7:            Select joint actions $u_{t}$ of UAVs based on policy $\pi_{\theta_{i}}\left(o_{t}^{i}\right)$8:            Execute joint actions $u_{t}$9:            Receive the team reward $r_{t}$ and the new state $o^{\prime }$10:         Store trajectory v = $v=\{u_{t},o_{t},u_{t+1},r_{t}\}$ to RB.11:   **end for**12:   **if** $r_{\mathrm{sum}\;}>\max\left(PB_{\mathrm{low}\;},\bar{r}\right)$ **then**13:         Add v to PB.14:   **else**15:         Add v to NB.16:   **end if**17:     Update the actor and critic networks.18:     Update MINE and CLUB with every k episodes.19:**end for**


## 5. Simulation, Results and Analysis

In this section, we describe extensive simulations conducted to validate the performance of the proposed CER-MADDPG scheme in UAV computation offloading and edge–server resource allocation problems.

### 5.1. Simulation Environment and Parameter Setting

We simulated an MEC environment using Python, where MEC servers were allocated alongside roads and some edge servers moved randomly in the communication area. In the simulation, all UAVs selected angles and distances to move within the communication range and edge servers for offloading requests based on the CER-MADDPG method. The simulation was performed using Python on a PyTorch platform. All simulation parameters used are shown in Table 1 and Table 2, which were used in our simulation experiments. Referring to the works [14,30,31,32,33,34], similar values are set in our simulations.

The UAV’s computation frequency, $f_{uav}$ is set to 1.2 GHz. The task size, taskSize is randomly generated when adjusting parameters, with values ranging from 30 to 40 Mbits. The number of CPU cycles required to process one bit, denoted as *s*, is set to 1000 cycles/bit. The UAV’s initial battery level is set to 500,000 J. These values are based on typical UAV parameters.

In our model, the location of the edge servers changes dynamically, simulating the potential movement of servers over time in a post-disaster environment. We use a simple linear mobility model to simulate server movement, where the servers can randomly select paths within a predefined area. The server’s movement speed is set to 1 m/s, and the server’s computation frequency, $f_{ue}$ is set to 5 GHz. This model can be further improved in the future with more complex random walk models to adapt to more complicated environmental changes.

### 5.2. Results and Analysis

#### 5.2.1. Selection of Hyperparameters

Setting the hyperparameters is crucial in the CER-MADDPG algorithm, because different hyperparameters can affect the optimization, convergence, and stability of the algorithm. Experiments were conducted to determine the optimal hyperparameters for the proposed algorithm.

**Figure 5:**
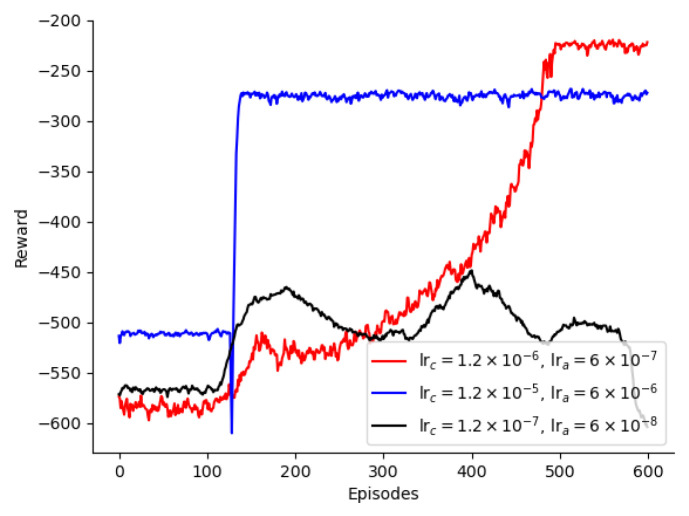
Selection of learning rates for the critic and actor networks.

In the CER-MADDPG algorithm, the learning rates of the actor and critic networks influence the training and updating of the neural networks. A high learning rate can lead to poor optimization and stability, whereas a low learning rate can result in poor optimization and slow convergence. In general, because the critic guides the update of the actor, the critic’s learning rate should be set slightly higher than that of the actor to ensure faster convergence. As shown in Figure 5, when the critic network’s learning rate is $1.2\times 10^{-5}$ and that of the actor network is $6\times 10^{-6}$, the reward increases with the number of iterations. However, the optimal solution here is not as good as that with a critic network’s learning rate of $1.2\times 10^{-6}$ and the actor network’s learning rate is $6\times 10^{-7}$. This is because a high learning rate causes both the critic and actor networks to update significantly, whereas the optimal solution requires smaller updates. When the learning rate for the critic network is $1.2\times 10^{-6}$ and that for the actor network is $6\times 10^{-7}$, the system’s cost can achieve a converged and stable optimal solution. Although a locally optimal solution was obtained after 170 rounds of training, a globally optimal solution could still be achieved by increasing the number of iterations. When the learning rate of the critic network is $1.2\times 10^{-7}$ and that for the actor network’s learning rate is $6\times 10^{-8}$, the system reward cannot reach the optimal solution, as the lower learning rates result in slower neural network updates, requiring more iterations to converge. Therefore, in this study, the optimal learning rate for the critic network is $1.2\times 10^{-6}$, and that for the actor network is $6\times 10^{-7}$. Additionally, it can be observed from the figure that after 100 rounds there was a significant change in the system cost. This is because at this point, the experience replay buffer is full, and the neural networks have sufficient useful information for training.

**Figure 6:**
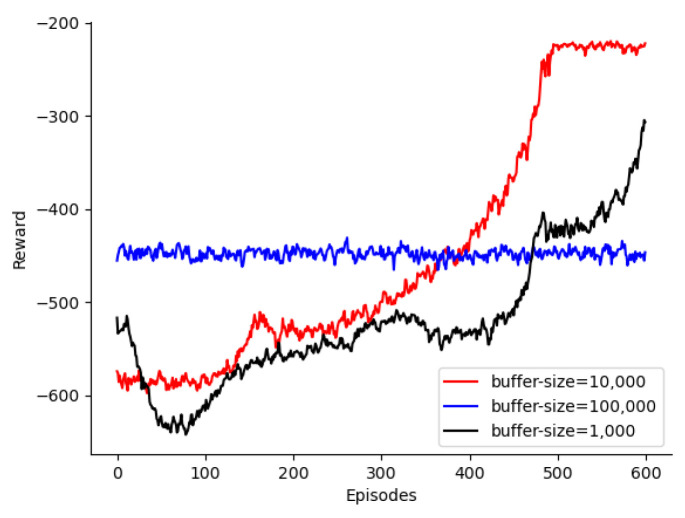
Selection of replay buffer size.

The experience replay buffer affected the training time, optimality, and convergence of the algorithm. As shown in Figure 6, a local optimal solution was obtained with an experience replay buffer size of 1000 and 320 training rounds; however, a global optimal solution could not be achieved. This is because a small experience replay buffer can affect the extraction of data features, leading to an inability to learn the optimal policy. When the experience replay buffer was increased to 10,000, a globally optimal solution was achieved after 480 rounds. However, with an experience replay buffer of 100,000, the optimal solution could not be obtained, because the buffer was too large and the data could not be updated, thus preventing the attainment of the best solution. Additionally, the algorithm with an experience replay buffer size of 10,000 trains was three times faster than that with an experience replay buffer size of 100,000. Therefore, we selected an optimal experience replay buffer size of 10,000.

**Figure 7:**
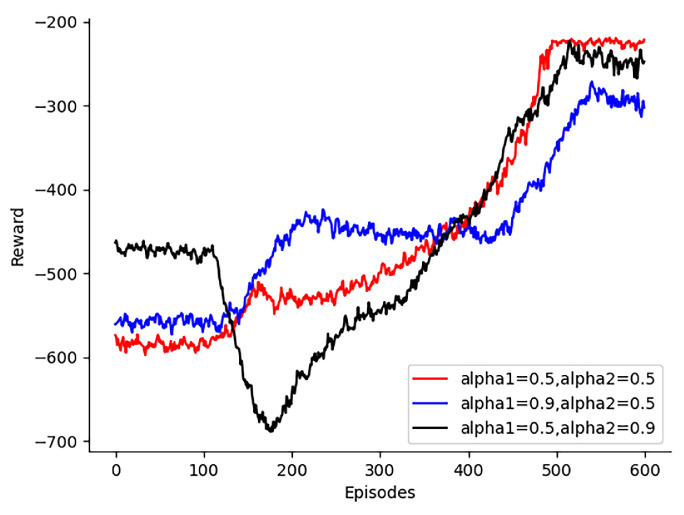
Selection of $\alpha_{1}$ and $\alpha_{2}$.

The selection of the mutual information parameters $\alpha_{1}$ for the lower bound of the high-quality experience buffer and $\alpha_{2}$ for the upper bound of the poor-quality experience buffer is shown in Figure 7. The choice of mutual information parameters affects the optimality and stability of the algorithm. When $\alpha_{1}$ is chosen as 0.5 and $\alpha_{2}$ is chosen as 0.5, the system reward converges steadily to the optimal solution. When $\alpha_{1}$ is chosen as 0.9 and $\alpha_{2}$ is chosen as 0.5, the system converges to a local optimal solution after 220 rounds. When $\alpha_{1}$ is chosen as 0.5 and $\alpha_{2}$ is chosen as 0.9, the system reward first decreases and then increases, and there is no stable convergence to the optimal solution. This is because neither a dominant weight for the upper bound of the poor-quality experience buffer nor a dominant weight for the lower bound of the high-quality experience buffer leads to convergence to the optimal solution. We summarize the experimental results in Table 3.

Through comparative experiments, it was verified that the experience buffer size should be set to $10^{4}$, learning rate of the actor network should be set to $10^{-7}$, learning rate of the critic network should be set to $10^{-6}$, $\alpha_{1}$ should be set to 0.5, and $\alpha_{2}$ should be set to 0.5.

#### 5.2.2. Performance Comparison and Analysis

We compared the CER-MADDPG method with MADDPG and SGRA-PERs to demonstrate the effectiveness of our approach. Brief introductions to these two methods are presented below.

- MADDPG: A policy gradient algorithm in which each agent has actor and critic networks. The critic network can access the states and actions of other agents during training, whereas the actor network requires only its own information. Consequently, the critic network is trained centrally, whereas the actor network is executed in a distributed manner.
- SGRA-PERs: Utilizes a prioritized experience replay mechanism for each UAV’s experience buffer, allowing important but infrequent experiences to be repeatedly utilized for learning, thereby enhancing system utility.

**Figure 8:**
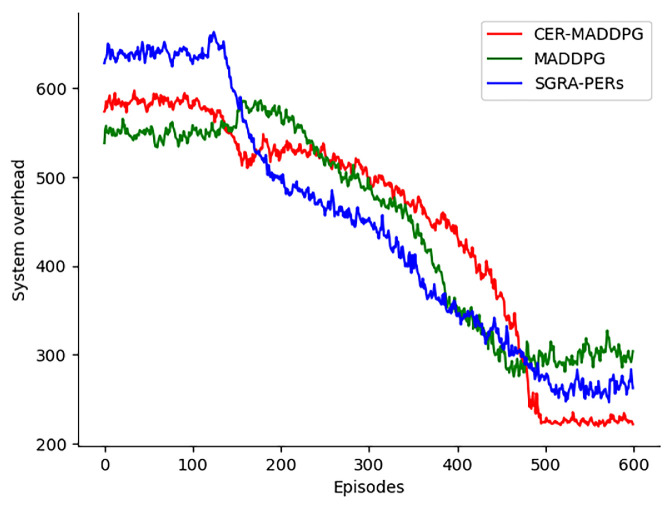
Comparison of system overhead of different algorithms.

As shown in Figure 8, with an increase in iterations, the proposed CER-MADDPG algorithm achieved the lowest system cost and optimal stability after 480 rounds. In comparison, the MADDPG algorithm achieved stable system utility after 460 rounds, and the SGRA-PERs achieved stable system utility after 520 rounds of training. However, the solutions obtained by these algorithms are locally optimal and do not achieve the best results. This is because the proposed CER-MADDPG considers the cooperation between agents more deeply and utilizes the classification of good and bad decision-making experiences of UAVs. The bidirectional guidance of joint decision-making by UAVs tends to exhibit good behavior and avoid bad behavior, thus achieving the best system utility. SGRA-PERs prioritizes historical experiences for each UAV, which leads to a lower system utility compared with that of MADDPG. It can guide UAVs to learn better strategies to some extent but still has limitations. Therefore, compared with other algorithms, the CER-MADDPG proposed in this study has advantages in terms of system cost and can obtain solutions with lower system costs.

**Figure 9:**
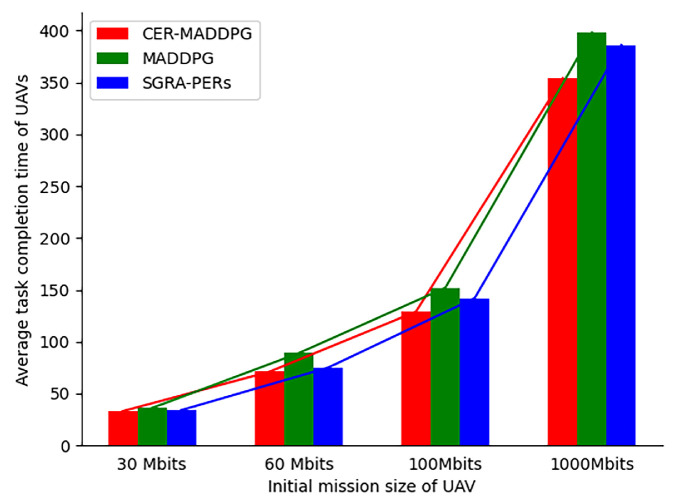
Comparison of task completion time for different UAV mission sizes.

To further validate the performance of the CER-MADDPG algorithm, Figure 9 illustrates the relationship between the initial task size of the UAVs and time required to complete the tasks. As the initial task size of the UAVs increases, the time required to complete the tasks also increases. Regardless of the initial task size, the CER-MADDPG required the shortest time to complete the tasks compared to those of the MADDPG and SGRA-PERs. Additionally, as the initial task size of the UAVs increased, CER-MADDPG exhibited the smallest increase in completion time, indicating higher stability than that of the MADDPG algorithm. This is because the CER-MADDPG algorithm proposed in this study fully utilizes good and bad experiences, enabling the UAV group to make more reasonable joint decisions and demonstrating advantages in stability and effectiveness.

**Figure 10:**
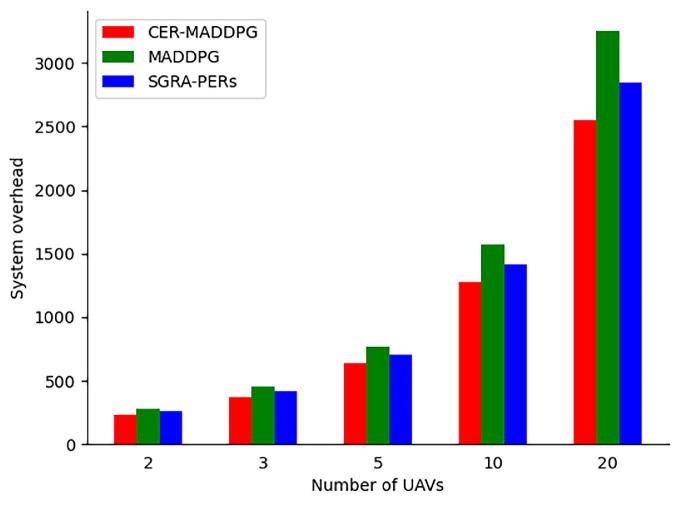
System consumption comparison as the number of UAVs increases.

We compared the system energy consumption of the four algorithms for different numbers of UAVs. We set the number of UAVs N to 2, 3, 5, 10, and 20. As shown in Figure 10, with an increase in the number of UAVs, the system costs of the different algorithms increase to some extent. The CER-MADDPG algorithm outperformed the other two algorithms in controlling system utility. This is because, as the number of UAVs increases, the total task processing time and energy consumption of the UAV group increase, resulting in an overall increase in system costs. The growth rate of CER-MADDPG was approximately constant, whereas those of MADDPG and SGRA-PERs were higher than that of CER-MADDPG. This is because CER-MADDPG utilizes an experience classification learning mechanism and considers both global and individual critic networks, enabling a more comprehensive consideration of the collaborative nature of joint decisions, thereby improving the system utility. In summary, the CER-MADDPG algorithm has significantly lower system costs than those of the other offloading methods, and this advantage increases with the number of UAVs. We summarize the experimental results in Table 4.

As shown in Table 4, irrespective of the number of UAVs, the system overhead of CER-MADDPG is smaller than those of SGRA-PERs and MADDPG. Additionally, from the changes in the system overhead, it can be observed that as the number of UAVs increases, the system overhead of the CER-MADDPG becomes more stable than those of the SGRA-PERs and MADDPG. Irrespective of the initial task of the UAVs (30 Mbits or 1000 Mbits), CER-MADDPG has the shortest average completion time for UAVs compared to those of the SGRA-PERs and MADDPG.

## 6. Conclusions

To address the limitations of UAV battery life and computing capabilities, this study considers a scenario with multiple UAVs and servers and proposes a UAV-assisted post-disaster rescue computation offloading scheme based on multi-agent reinforcement learning. The algorithm proposed herein delegates decision-making to UAVs, and is designed to consider cooperative decision-making among multiple UAVs. Additionally, the algorithm focuses on guiding UAVs to make decisions based on good experiences and avoid bad experiences, enabling them to make optimal joint offloading decisions and ultimately improving system performance. The simulation results demonstrate that the proposed CER-MADDPG algorithm achieves better effectiveness and stability in joint decision-making compared with SGRA-PERs and MADDPG, effectively reducing the system overhead. The CER-MADDPG can generate optimal strategies based on time-varying channel conditions and edge server states.

Fault tolerance in UAV edge computing systems [35] is a new field that must be explored in the future. In this study, the UAVs, channels, and edge devices were assumed to be honest. In future research, we will consider security issues, improve the existing models, and design a model that is more suitable for practical applications.

## Figures and Tables

**Table 1:** Related parameters of environment.

Parameters	Values
groundHeight	100 m
groundLength	100 m
groundWidth	100 m
$f_{ue}$	5 GHz
$f_{uav}$	1.2 GHz
*s*	1000 cycles/bit
$v_{ue}$	1 m/s
battery	500,000
taskSize	30–40 Mbits

**Table 2:** Related parameters of CER-MADDPG.

Parameters	Values
Critical neural network structure	GRU, MLP
GRU network layers	1
MLP network layers	The number of UAVs
Batch size	128
Positive buffer size	$10^{4}$
Negative buffer size	$10^{4}$
Soft update factor	0.1
Epsilon	0.1
$\lambda_{1}$	1
$\lambda_{2}$	1

**Table 3:** Hyperparameter settings.

Parameters	Values
Replay buffer size	$10^{4}$
Actor network learning rate	$6\times 10^{-7}$
Critic network learning rate	$1.2\times 10^{-6}$
$\alpha_{1}$	0.5
$\alpha_{2}$	0.5

**Table 4:** Simulation results.

Name	CER-MADDPG	SGRA-PERs	MADDPG
System overhead (2 UAVs)	232	257	282
System overhead (20 UAVs)	2554	2847	3250
Average task completion time of UAV (task size 30 Mbits)	33	34	36
Average task completion time of UAV (task size 1000 Mbits)	354	386	398

## Data Availability

No new data were created or analyzed in this study. Data sharing is not applicable to this article.
